# ASO Author Reflections: Cytology-Positive Gastric Cancer: Nodal Status Dominates Prognosis, HIPEC Awaits Validation

**DOI:** 10.1245/s10434-025-18950-x

**Published:** 2025-12-26

**Authors:** Vikram A. Chaudhari, Kunal Nandy, Amir Parray, Shailesh V. Shrikhande, Manish S. Bhandare

**Affiliations:** 1 Division of GI and HPB Surgery, Department of Surgical Oncology, Tata Memorial Hospital, Homi Bhabha National Institute, Mumbai, India; 2 HPB & Upper GI Unit, Department of Surgical Oncology, Tata Memorial Centre - Advanced Centre for Treatment, Research and Education in Cancer (ACTREC), Homi Bhabha National Institute, Mumbai, India

## Past

The management of isolated cytology-positive (Cy+) gastric cancer (PCI 0) has long been debated. Although it is classified as stage IV disease, Cy+ patients represent a biologically and clinically distinct subgroup: their outcomes are consistently worse than those of cytology-negative patients but markedly superior to those with overt peritoneal metastases.^1^ This intermediate phenotype has created a therapeutic grey zone, leading to heterogeneous strategies that range from systemic therapy alone to systemic therapy with gastrectomy and, in select centres, the addition of hyperthermic intraperitoneal chemotherapy (HIPEC). Existing literature is inconsistent, largely because of variations in patient selection, treatment protocols, and the intended role of HIPEC within multimodal therapy.^2,3^

## Present

In our institutional cohort of 558 patients who received neoadjuvant chemotherapy followed by curative-intent gastrectomy (2017–2023), isolated cytology positivity was identified in 47 patients (8.4%) with PCI 0 disease.^4^ Despite standardized surgery and perioperative systemic treatment, Cy+ status independently conferred a two-fold increase in mortality risk compared with cytology-negative patients (hazard ratio [HR] 2.1; 95% confidence interval [CI] 1.4–3.2). Within this high-risk subgroup, nodal metastasis emerged as the principal determinant of outcome, tripling the risk of recurrence (HR 3.2; 95% CI 1.6–6.4) and surpassing cytology status itself in prognostic weight. These findings challenge the prevailing assumption that prognosis in Cy+ disease is driven chiefly by peritoneal biology and instead highlight nodal burden as a marker of systemic tumor dissemination that cannot be mitigated by locoregional therapy alone.

Almost half of Cy+ patients (n = 23; 48.9%) underwent HIPEC. The procedure was feasible, with no 90-day mortality and similar major morbidity to surgery alone. However, HIPEC did not confer meaningful oncologic benefit. Median overall survival was 42 versus 38 months (p = 0.18), and median disease-free survival was 24 versus 20 months (p = 0.24). The absence of significant improvement, despite acceptable perioperative outcomes and adequate follow-up, reinforces that intraperitoneal chemotherapy cannot compensate for unfavorable systemic biology. These observations align with emerging evidence that peritoneal-directed therapy may be insufficient in Cy+ disease with nodal involvement and should not be applied indiscriminately.

## Future

These findings support a transition from routine locoregional intervention to biologically informed treatment selection in Cy+ gastric cancer. Future trials evaluating HIPEC should stratify patients according to nodal status, post-neoadjuvant cytology conversion, and molecular characteristics rather than treating Cy+ disease as a homogeneous entity. Biomarker-guided approaches—including microsatellite instability, programmed cell death ligand-1 expression, and circulating tumor DNA—may identify subgroups that are more likely to benefit from peritoneal-directed therapy.

Standardization of peritoneal cytology techniques, including immunohistochemical or molecular characterization of isolated tumor cells, will be critical to refining risk assessment. The clinical value of cytology conversion after systemic therapy remains insufficiently defined and may help identify chemotherapy-responsive patients who do not require additional locoregional therapies.

Until high-quality randomized data—such as the results of GASTRICHIP—are available, HIPEC remains investigational.^5^ Current evidence supports prioritization of oncologic fundamentals: R0 resection with adequate lymphadenectomy and optimized perioperative systemic therapy. Management should be driven primarily by biological risk, particularly nodal burden, rather than routine incorporation of peritoneal-directed therapy.
